# Association between polymorphisms in the coagulation factor VII gene and coronary heart disease risk in different ethnicities: a meta-analysis

**DOI:** 10.1186/1471-2350-12-107

**Published:** 2011-08-12

**Authors:** Xingbo Mo, Yongchen Hao, Xueli Yang, Shufeng Chen, Xiangfeng Lu, Dongfeng Gu

**Affiliations:** 1 Department of Evidence Based Medicine and Division of Population Genetics, Cardiovascular Institute and Fuwai Hospital, Chinese Academy of Medical Sciences and Peking Union Medical College, Beijing, China

**Keywords:** Coagulation factors, FVII, Polymorphisms, Coronary heart disease, Meta-analysis

## Abstract

**Background:**

Previous studies have examined the association between polymorphisms in the coagulation factor VII gene and the risk of coronary heart disease (CHD), but those studies have been inconclusive. This study was conducted to assess the associations between these polymorphisms and CHD and evaluated the associations in different ethnicities.

**Methods:**

Literature-based searching was conducted to collect data and two methods, namely fixed-effects and random-effects, were performed to pool the odds ratio (OR), together with the 95% confidence interval (CI). Publication bias and between-study heterogeneity were also examined.

**Results:**

Thirty-nine case-control studies of the three polymorphisms, R353Q (rs6046), HVR4 and -323Ins10 (rs36208070) in factor VII gene and CHD were enrolled in this meta-analysis, including 9,151 cases of CHD and 14,099 controls for R353Q, 2,863 cases and 2,727 controls for HVR4, and 2,862 cases and 4,240 controls for -323Ins10. Significant association was only found in Asian population for R353Q (Q vs R), with pooled OR of 0.70(95%CI: 0.55, 0.90). For the -323Ins10 polymorphism (10 vs 0), we found significant associations in both Asian and European populations, with pooled ORs of 0.74(95%CI: 0.61, 0.88) and 0.63(95%CI: 0.53, 0.74), respectively. Marginal significant association was found between HVR4 (H7 vs H5+H6) and CHD (OR = 0.88, 95% CI: 0.78, 1.00). There was no evidence of publication bias, but between-study heterogeneity was found in the analyses.

**Conclusions:**

The -323Ins10 polymorphism in factor VII gene is significantly associated with CHD in both Asian and European populations, while R353Q polymorphism showed trend for association with CHD in Asians. Lack of association was found for HVR4 polymorphism. Further studies are needed to confirm the association, especially for -323Ins10 polymorphism.

## Background

Coronary heart disease (CHD) is the most common cause of death in industrialized countries, and is rapidly increasing in prevalence in developing countries such as China and India. CHD is a multifactorial disease influenced by both genetic and environmental determinants. Coagulation activation plays a key role in thrombus formation and variation and its factors have been associated with the risk of CHD. Activation of the extrinsic coagulation pathway plays a key role in hemostasis, and thus factor VII contributes to the occurrence of thrombotic events. High factor VII levels might disproportionately enhance the coagulation cascade at the time of plaque rupture, which could explain the apparently differential association with fatal and nonfatal events.

There have been a number of reports that increased factor VII activity is a risk factor for CHD [1-3], and it is now well documented that certain polymorphisms in the factor VII gene are strongly associated with levels of Factor VIIc, Factor VIIa and Factor VII antigen [1,4,5]. The R353Q (rs6046) and -323Ins10 polymorphisms (rs36208070) have been associated with a 20% to 25% reduction in plasma factor VII levels [6]. The R353Q polymorphism alone can decrease plasma factor VII levels [7]. Significant associations between R353Q and -323Ins10 polymorphisms and FVII coagulant activity (FVIIc) and antigen (FVIIAg) levels have also been reported in Chinese population [8]. A recent genome wide association study (GWAS) identified a SNP (rs488703, in perfect LD with R353Q (rs6046)) in coagulation factor VII gene significantly associated with plasma levels of coagulation factor VII (p-value = 9.0*10^-259^) [9]. Previous studies have examined the association of the three polymorphisms in the factor VII gene, including R353Q polymorphism, intron 7 polymorphism (HVR4:H5, H6 and H7 alleles), and -323 0/10 bp insertion/deletion polymorphism (allele A1 corresponds to the absence of the decamer (0) and allele A2 to its insertion 10), with the risk of CHD. But those studies have been inconclusive.

For the R353Q polymorphism, a single nucleotide change determines the presence of glutamine (Q) or arginine (R) at amino acid 353 of factor VII. The HVR4 polymorphism is a variable tandem repeat and consists of three alleles: H5, H6 and H7. Some studies concluded a protective role for the QQ and H7H7 genotypes in the risk of myocardial infarction (MI) among patients with CHD [10-12]. However, others failed to confirm such results [13-16], including the Framingham Heart study [17].

The -323Ins10, a 10-bp insertion in the promoter region in linkage disequilibrium with R353Q [8,18], also has been demonstrated to be functionally relevant; the rare insertion allele of 10 bp, indeed, reduced the promoter activity, as compared with the common allele, in transfection experiments [19]. There have been several reports on the association of cardiovascular disease with this polymorphism (see Additional file 1). However, the results have been conflicted.

Meta-analysis is a powerful tool for summarizing results from different studies by producing a single estimate of the major effect with enhanced precision. However, previous meta-analyses performed on studies for factor VII (R353Q polymorphism) got different results [20,21]. In this study, we conducted a meta-analysis to examine the associations between the three polymorphisms in the factor VII gene and CHD, and evaluated the associations in different ethnicities. We report an updated meta-analysis for the R353Q polymorphism as well as new pooled results for two polymorphisms (HVR4 and -323Ins10).

## Methods

### Retrieval of published studies

We searched information from HuGE navigator (http://www.hugenavigator.net/), and conducted a systematic computerized literature search for papers published before November 23, 2010. We searched PubMed, Embase and two Chinese databases, Cqvip and CBM, using various combinations of keywords, such as 'coronary heart disease' or 'coronary artery disease' or 'myocardial infarction' combined with 'FVII' or 'coagulation factor VII' and 'polymorphism' and 'genetic association'. Manuscripts in languages of Chinese and English were considered for review. The full texts of the retrieved articles were read to decide whether information on the topic of interest should be included. The reference lists of the retrieved articles as well as those of review articles and previous meta-analyses on this topic were searched to identify other studies that were not identified initially. Articles were included in the meta-analysis if they examined the hypothesis that factor VII was associated with CHD using a case-control design, and had sufficient published data on the genotypes or allele frequencies for determining an estimate of relative risk (i.e. odds ratio (OR)) and a confidence interval (CI).

### Data extraction

Two reviewers (Mo and Yang) independently examined the retrieved articles using a data collection form, in order to extract the information needed. From each study the following data were abstracted: first author's last name, year of publication, country of the population studied, the counts of persons with different genotypes in cases and controls, the average age and the percentage of men in case and control groups within each study. Following data extraction, the reviewers checked for any discordance until a consensus was reached.

### Statistical analysis

The OR was used to compare contrasts of alleles or genotypes between cases and controls. We computed the genetic contrast of the mutant alleles (Q for R353Q, H7 for HVR4 and insertion 10 for -323 0/10 bp insertion/deletion polymorphism) versus the wildtype alleles and contrasts of homozygous genotypes against the others. In secondary analyses, we calculated specific ORs according to the racial descent of subjects (separated analyses for Caucasian, East Asian and other population). We assessed the presence of between-study heterogeneity using the chi-square based Cochran's Q statistic. The inconsistency index I^2 ^(ranging from 0 to 100%) was also calculated, where higher values of the index (I^2 ^> 50%) indicate the existence of heterogeneity [22]. Publication bias was assessed with funnel plots and Egger regression test [23]. The combined ORs along with their 95% CIs were estimated using the fixed-effects or random-effects method. The random-effects method [24], which in the presence of heterogeneity, is more appropriate as it is prudent to take into account an estimate of the between-study variance (I^2^). Deviations from the HWE were calculated by the chi-square method. To examine specific subsets in these studies, separate analyses were undertaken. This was achieved by performing a sensitivity analysis, in which an individual study was removed each time to assess the influence of each study. Likewise, a cumulative analysis was performed according to the ascending date of publication to identify the influence of the first published study on the subsequent publications and the evolution of the combined estimate over time [25]. For all analyses performed here, the statistical package Stata 10 (Stata Corporation, College Station, Texas, USA) and the *catmap *package developed using the R language (http://www.r-project.org) were used. In all analyses statistically significant results were declared as those with a P value < 0.05.

## Results

**Figure 1:**
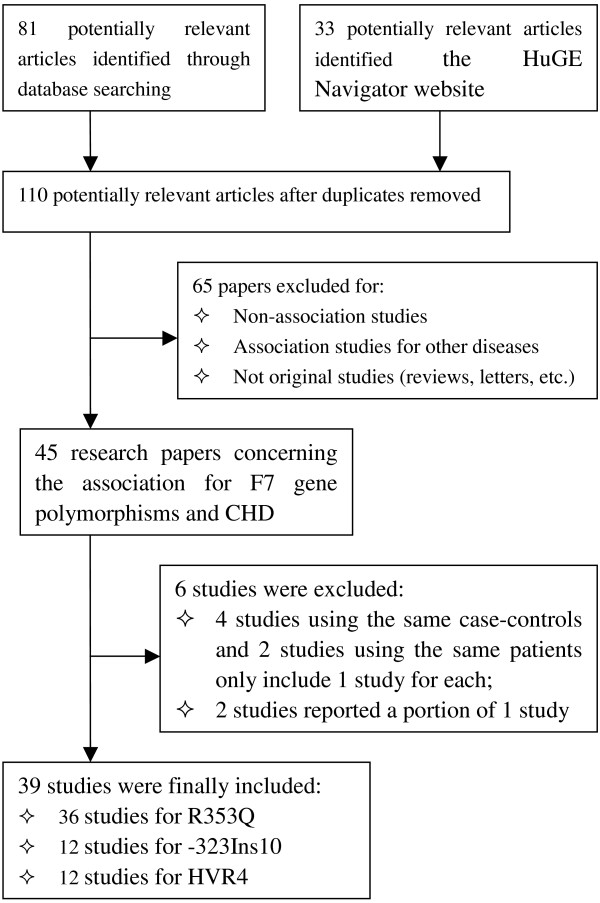
**Flow diagram for study selection process in the meta-analysis of FVII gene polymorphisms and CHD**.

We found 33 articles related to this topic on the HuGE Navigator website. After the literature search and the subsequent screening, we came up with 45 research papers concerning the association of R353Q or -323Ins10 or HVR4 polymorphism with CHD. One of these papers contained information about distinct independent populations and thus they are considered as different studies which should be counted twice [26]. Other papers used the same case-control groups [26-29], the same CHD patients [30,31], or reported different proportions according to different populations or polymorphisms [32,33] of one study [26]. These studies were used only once and participated in the sensitivity analysis. A total of 39 studies were finally included in this study (Figure 1). The detailed characteristics of each study (Authors, year, and mean age, percentage of men and genotype data in case control groups, etc.) were summarized (see Additional file 1).

### Meta-analysis for R353Q polymorphism

The 36 retrieved studies that were investigating the association of R353Q polymorphism with CHD contained information about 9,151 cases and 14,099 control subjects (Table 1). For four of these 36 studies it is apparent that deviations from hardy-Weinberg equilibrium (HWE) could be detected. The pooled frequency of the Q allele in CHD cases and controls was 12.2% and 12.8% separately, and it was found to be 14.2% and 8.8% in the control subjects of Caucasian populations and Asians, respectively.

**Table 1:** Meta-analysis of FVII gene polymorphisms and CHD

Polymorphisms	Populations	Number of studies	Number of cases	Number of controls	MAF %	OR(95%CI)	P -value	I^2^ %
R353Q	European	20	5720	10641	14.2	1.02(0.94-1.11)	0.623	11.4
(rs6046)	East Asian	12	2652	2654	8.8	0.70(0.55-0.90)	0.005	55.5
	Other	4	779	804	32.6	0.92(0.78-1.07)	0.280	0.0
	**Total**	**36**	**9151**	**14099**	**12.8**	**0.91(0.83-1.01)**	**0.069**	**51.2**
								
-323Ins10	European	5	926	2426	13.0	0.74(0.61-0.88)	0.001	35.1
(rs36208070)	East Asian	5	1620	1476	13.6	0.63(0.53-0.74)	< 0.001	0.0
	Other	2	316	338	35.8	1.10(0.84-1.43)	0.500	0.0
	**Total**	**12**	**2862**	**4240**	**17.3**	**0.73(0.66-0.82)**	**< 0.001**	**45.2**
								
HVR4	European	4	611	560	32.8	0.82(0.55-1.21)	0.317	76.6
	East Asian	6	1793	1711	42.3	0.87(0.75-1.01)	0.059	47.0
	Other	2	459	456	30.1	0.97(0.68-1.38)	0.844	65.6
	**Total**	**12**	**2863**	**2727**	**38.3**	**0.88(0.78-1.00)**	**0.059**	**57.4**

MAF = Minor Allele Frequency (in control groups); OR = Odds Ratio; CI = Confidence Interval;

I^2^: The inconsistency index for between-studies heterogeneity, where higher values of the index (I^2 ^> 50%) indicate the existence of heterogeneity.

**Figure 2:**
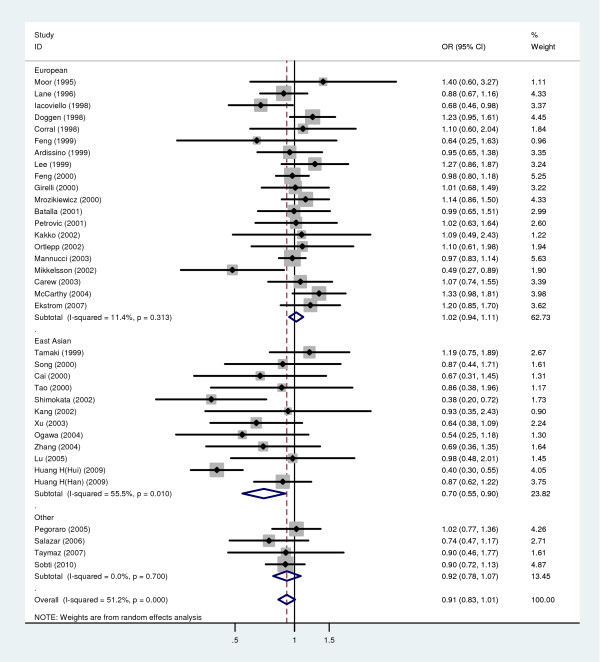
**Meta-analysis for the relationship between R353Q polymorphism and CHD risk**. The combined ORs along with their 95% CIs were in the contrast of Q allele vs. R allele and estimated using the random-effects method.

Figure 2 shows that the combined ORs (random-effects method) for the Q allele on CHD risk were 1.02 (95% CI: 0.94, 1.11) in European population and 0.70 (95%CI: 0.55, 0.90) in Asian population. There was a significant between-study heterogeneity as indicated by the P value of the corresponding test (P = 0.01) and the value of the I^2 ^index (I^2 ^= 55.5%) for studies in Asian population. The sensitivity analysis revealed the evidence for a single influential study that was biasing the results. This study was that of Huang's research on the Chinese Ningxia Hui population [26]. If this study was removed the analysis would produce an overall estimate of 0.93 (95% CI: 0.86, 1.01). In the cumulative meta-analysis we also observed the influential role of the study by Huang et al. [26]. The overall estimation became significant only after this study was included in the cumulative meta-analysis. We assessed publication bias using formal statistics, Begg's test and Egger's test. The results did not suggest publication bias in the studies (Begg's test, P = 0.212, and Egger's test, P = 0.213)

Similar results were observed in a dominant model. We did not examine a recessive model because the number of QQ genotype in both cases and controls of six studies equals to zero. Furthermore, significant between-study heterogeneity was not found after excluding studies not in HWE, but this did not influence the results significantly.

### Meta-analysis for -323Ins10 polymorphism

Twelve studies investigated the association of -323Ins10 polymorphism with CHD, including 2,862 cases and 4,240 controls, were enrolled in this meta-analysis (Table 1). For all the studies included, we found that the control groups were all in HWE. The pooled frequency of the A2 allele in CHD cases and controls was 14.7% and 17.3%, respectively.

**Figure 3:**
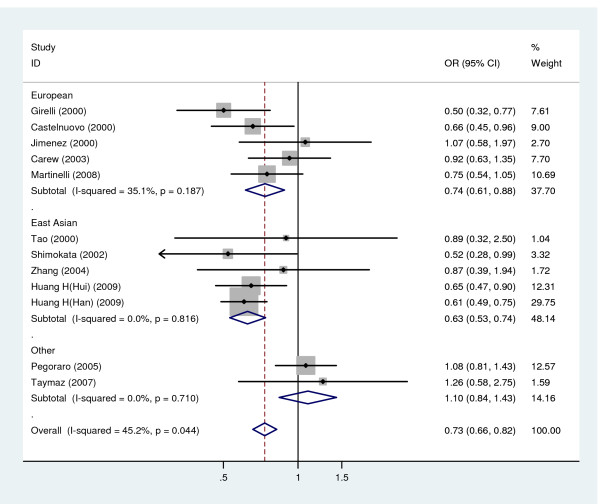
**Meta-analysis for the relationship between -323Ins10 polymorphism and CHD risk**. The combined ORs along with their 95% CIs were in the contrast of A2 allele vs. A1 allele and estimated using the fixed-effects method.

A significant association was observed between the A2 allele and the risk for CHD (Figure 3), yielding an overall OR (fixed-effects method) of 0.73 (95% CI: 0.66, 0.82). A dominant model also yielded a similar result, with a significant overall OR of 0.69 (95% CI: 0.60, 0.78). By performing subgroup analyses we found significant associations in both Caucasian and Asian groups (Figure. 3). In the two contrasts considered (A2 allele versus A1 allele and A2A2+A1A2 versus A1A1), no significant heterogeneity was observed (P = 0.047 and 0.278, I^2 ^= 45.2 and 24.9%, respectively). The sensitivity analysis revealed that there was not a single study influencing the results significantly. After removing each study and calculating the overall estimate and the 95% CI for the remaining studies, the significance of the OR remained unchanged. There was no publication bias in the studies.

### Meta-analysis for HVR4 polymorphism

Twelve studies including 2,863 cases and 2,727 controls were enrolled in this meta-analysis for the association between HVR4 polymorphism and CHD (Table 1). The control groups for all the studies included were in HWE. The pooled frequency of the H7 allele was 35.3% and 38.3% in CHD cases and controls, respectively.

**Figure 4:**
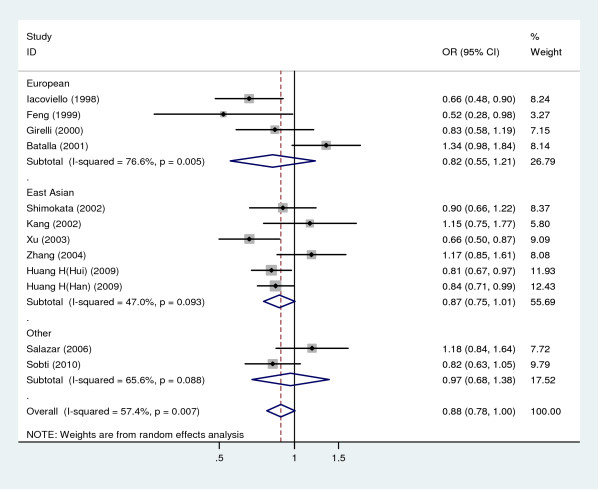
**Meta-analysis for the relationship between HVR4 polymorphism and CHD risk**. The combined ORs along with their 95% CIs were in the contrast of H7 allele vs. H5+H6 allele and estimated using the random-effects method.

The contrast of the H7 allele versus H5+H6 allele was examined, marginal significant association was found between the HVR4 polymorphism and the risk for CHD (OR = 0.88, 95% CI: 0.78, 1.00, random-effects method) (Figure 4). Performing subgroup analyses we found that European studies indicated no significant association (OR = 0.82, 95% CI: 0.55, 1.21). The combined OR for the studies involving Asian subjects was 0.87 (95% CI: 0.75, 1.01), as well as homogeneity of the results (I^2^= 47.0%, P = 0.093) (Figure 4). There was a statistically significant heterogeneity for the overall estimates as indicated by the P value (equals to 0.007) of the corresponding test and I^2 ^= 57.4%. The sensitivity analysis revealed that there was not a single study influencing the results significantly. In the cumulative meta-analysis, we also observed strong evidence suggesting that the first published study that reported a significant association [11]. The overall estimation became insignificant only after the study reported by Batalla [34] was included in the cumulative meta-analysis. The results of Begg's test and Egger's test did not suggest publication bias in the studies (Begg's test, P = 0.537, and Egger's test, P = 0.635).

## Discussion

A controversial relationship was reported between the polymorphisms within the coagulation factor VII gene and CHD in different populations, including previous meta-analyses, led us to conduct the present study.

Wu et al.[20] conducted a meta-analysis and reported that the combined RQ and QQ genotype of factor VII R353Q was correlated to a reduced risk for cardiovascular disease in 2,574 patients (OR 0.78, 95% CI 0.65 to 0.93), whereas the QQ genotype had offered more protection (OR 0.53, 95% CI 0.27 to 1.03). However, the combined analyses result reported by Ye and coworkers [21], among 24 available studies with a total of 7,444 patients and 12,110 control individuals, showed no significant overall associations with CHD, yielding per-allele relative risks (RR) of 0.97 (95% CI 0.91 to 1.04). Our study showed the significant association between R353Q polymorphism and CHD in Asian population. The most noteworthy findings of this meta-analysis were that the 353QQ+QR genotype and 353Q allele consistently appeared to be significantly associated with a reduced risk of CHD. Our result for the overall population supported that of the recent meta-analysis conducted by Ye and coworkers. We found five studies reported after 2006, when Ye et al. conducted their meta-analysis, and we reviewed these studies in our study. In cumulative analysis we found significant overall associations between the R353Q polymorphism and the risk for CHD only after the study published by Huang et al. [26] was included. Nevertheless, we still failed to find significant associations for European and other populations.

For the -323Ins10 polymorphism, the A2 allele was observed to be a protective effect in both Asian and European individuals, without publication bias and significant heterogeneity. The A2A2+A2A1 genotype and A2 allele consistently appeared to be significantly associated with a reduced risk of CHD. For the HVR4 polymorphism, we also found a protective effect in Asian individuals, although the odds ratio failed to reach statistical significance in European population. The study samples for the -323Ins10 and HVR4 polymorphism were still small, further large case-control studies are needed for these two polymorphisms.

According to our results, the association between F7 polymorphisms and CHD risk may be different in different ethnicities. Significant association was only found in East Asian population for the R353Q polymorphism in our meta-analysis. We found no significant associations in the Europeans, this result was consistent with the study of CARDIoGRAM consortium (by corresponding to authors). The -323Ins10 polymorphism was significantly associated with CHD risk in both European and East Asian populations. Lack of association was found for HVR4 polymorphism both in European and East Asian populations.

Several potential limitations of our study should be noted. First, we should realize that the results might be distorted by potential weakness and biases of genetic association studies, such as genotyping error, phenotype misclassification, population stratification, gene-gene or gene-environment interactive effect, and selective reporting biases [25,35]. Second, although no statistically significant publication bias was found from Egger's test, exclusion of unpublished studies may affect the validity of the analysis. The eligible studies in our research were mainly from Asia and Europe, data of other populations, like African, was limited. Third, because we did not have access to individual data, we could not control for population stratification, nor could we adjust for variables in possible intermediate pathways.

The exact biological role of the particular polymorphism remains to be determined. In theory, FVII contribute to atherosclerosis through the generation of thrombin and fibrin formation. Development of coagulation in the vessel wall may result in production of thrombin and activation of platelet, leading to the release of various cytokines and the proliferation of smooth muscle cells in the vessel wall. Thus, someone with a lower FVIIc level may have less chance of developing CAD. Moreover, thrombin can have pro-inflammatory properties and pro-angiogenic activities. Vascular tone, inflammation, blood viscosity, and angiogenesis can play roles in initiating and maintaining an elevation in BP and, therefore, are potential mechanisms contributing to the association between FACTOR VII and risk factors like smoking, diabetes, hypertension, and mental stress. The FVII polymorphisms were reported to be associated with decreased blood pressure and a decreased risk of hypertension [36], suggesting that these FVII variants might influence cardiovascular risk through mechanisms other than thrombosis.

Further studies which aim to confirm the functional variants should be needed. More studies are needed to elucidate the complete range of the signal transduction pathways that the variant is implicated in, and thus, throw light in the underlying molecular mechanisms that confer susceptibility to CHD. The particular polymorphism associated with CHD itself may not play a functional role, but rather it may be located physically close to the actual disease-predisposing gene.

In addition to the genetic markers described in this analysis, many other polymorphisms in genes encoding important biochemical or coagulation factors have been studied. Those polymorphisms may be candidates for a multivariate analysis. Future haplotypic approaches and further haplotype-based meta-analyses will provide more powerful and informative results than current single genotype-based data.

## Conclusions

The results of the present research suggested that -323Ins10 polymorphism is significantly associated with CHD. R353Q polymorphism showed trend for association with CHD in Asian people but probably not in Europeans. Lack of association was found for HVR4 polymorphism. Further studies are needed to confirm the association, especially for -323Ins10 polymorphisms.

## Abbreviations

CHD: coronary heart disease; CI: confidence interval; FVII: coagulation factor VII; HuGE: human genome epidemiology; HWE: hardy-Weinberg equilibrium; MI: myocardial infarction; OR: odds ratio.

## Competing interests

The authors declare that they have no competing interests.

## Authors' contributions

XBM and DFG conceived of the study, and participated in its design and coordination. XBM, YCH and XLY carried out the literature searching and data extraction, independently. XBM and YCH performed the statistic analysis. SFC and XFL help to draft the manuscript. All authors read and approved the final manuscript.

## Pre-publication history

The pre-publication history for this paper can be accessed here:

http://www.biomedcentral.com/1471-2350/12/107/prepub

## Supplementary Material

Additional file 1 **Characteristics of the 39 eligible studies included in meta-analysis**. A table summarized the detailed characteristics of each study included in the meta-analysis (First authors, published year, and mean age, percentage of men and genotype data in case control groups, etc.). ^a ^323 in the 5' promoter region, (10-bp insertion in the promoter region (5'FVII)), where allele *A1 *corresponds to the absence of the decamer (0) and allele *A2 *to its insertion(10). ^b ^NS = not specified, M = matched. ^c ^Study for Chinese Hui population. ^d ^Study for Chinese Han population Click here for file
