# Supplementary material for: Association between polymorphisms in the coagulation factor VII gene and coronary heart disease risk in different ethnicities: a meta-analysis
Source: BMC Med Genet. 2011 Aug 12;12:107. doi: 10.1186/1471-2350-12-107 (PMC3166910; doi:10.1186/1471-2350-12-107)
Supplement: Additional file 1 — Characteristics of the 39 eligible studies included in meta-analysis. A table summarized the detailed characteristics of each study included in the meta-analysis (First authors, published year, and mean age, percentage of men and genotype data in case control groups, etc.). a 323 in the 5' promoter region, (10-bp insertion in the promoter region (5'FVII)), where allele A1 corresponds to the absence of the decamer (0) and allele A2 to its insertion(10). b NS = not specified, M = matched. c Study for Chinese Hui population. d Study for Chinese Han population [file 1471-2350-12-107-S1.DOC]

Additional file 1 -Characteristics of the 39 eligible studies included in meta-analysis

| **study** | **ethnicity** | **Year** | **Mean age** | | **Percentage of men (%)** | | | **R353Q**  **(case/control)** | | | **HVR4**  **(case/control)** | | | | | **-323Ins10^a^**  **(case/control)** | | |
| --- | --- | --- | --- | --- | --- | --- | --- | --- | --- | --- | --- | --- | --- | --- | --- | --- | --- | --- |
|  |  |  | Case | Control | Case | Control | | RR | RQ | QQ | H77 | H76 | H66 | H65 | H75 | A1A1 | A1A2 | A2A2 |
| Moor | European | 1995 | 39.6 | 40.2 | NS^b^ | | NS | 82/89 | 11/10 | 1/0 |  |  |  |  |  |  |  |  |
| Lane | European | 1996 | M^b^ | M | M | | M | 370/483 | 88/129 | 3/6 |  |  |  |  |  |  |  |  |
| Iacoviello | European | 1998 | 55.0 | 56.0 | 78.0 | | 68.0 | 114/138 | 49/76 | 1/10 | 12/31 | 60/97 | 84/94 | 5/2 | 4/1 |  |  |  |
| Doggen | European | 1998 | 56.2 | M | 100 | | NS | 440/529 | 115/111 | 5/4 |  |  |  |  |  |  |  |  |
| Corral | European | 1998 | 62.9 | M | 73.3 | | M | 78/80 | 22/20 | 1/1 |  |  |  |  |  |  |  |  |
| Feng | European | 1999 | 54.0 | 53.0 | 68.0 | | 44.0 | 81/18 | 19/7 | 0/0 | 7/5 | 44/12 | 49/8 | 0/0 |  |  |  |  |
| Ardissino | European | 1999 | 40.7 | 41.3 | 93.0 | | 93.0 | 143/139 | 51/56 | 6/5 |  |  |  |  |  |  |  |  |
| Lee | European | 1999 | 71.1 | 68.8 | 57.9 | | 49.6 | 148/327 | 27/56 | 9/11 |  |  |  |  |  |  |  |  |
| Girelli | European | 2000 | 61.3 | 57.5 | 87.6 | | 51.3 | 197/94 | 81/34 | 7/5 | 19/15 | 70/48 | 78/45 | 3/1 | 1/1 | 132/64 | 38/39 | 5/7 |
| Castelnuovo | European | 2000 | 55.0 | 56.0 | 78.0 | | 67.0 |  |  |  |  |  |  |  |  | 129/144 | 46/78 | 1/ 5 |
| Jimenez | European | 2000 | 48.0 | 51.0 | 78.4 | | 74.1 |  |  |  |  |  |  |  |  | 88/86 | 22/22 | 1/0 |
| Feng | European | 2000 | 70.4 | 56.1 | 53.0 | | 45.0 | 385/1986 | 119/643 | 12/59 |  |  |  |  |  |  |  |  |
| Mroziklewicz | European | 2000 | NS | NS | NS | | NS | 257/791 | 70/179 | 4/14 |  |  |  |  |  |  |  |  |
| Batalla | European | 2001 | 41.0 | 42.0 | 100 | | 100 | 130/154 | 43/38 | 2/8 | 23/20 | 68/66 | 82/108 | 2/6 |  |  |  |  |
| Petrovic | European | 2001 | 51.0 | 52.0 | 90.0 | | 82.0 | 124/98 | 41/33 | 2/1 |  |  |  |  |  |  |  |  |
| Kakko | European | 2002 | 52.8 | 52.8 | 86.0 | | 86.0 | 129/130 | 13/12 | 0/0 |  |  |  |  |  |  |  |  |
| Ortlepp | European | 2002 | 58.0 | 59.1 | 68.0 | | 68.0 | 76/78 | 22/20 | 2/2 |  |  |  |  |  |  |  |  |
| Mikkelsson | European | 2002 | NS | NS | 100 | | NS | 57/103 | 15/57 | 0/3 |  |  |  |  |  |  |  |  |
| Mannucci | European | 2003 | 39.0 | M | 87.7 | | M | 869/863 | 321/325 | 20/22 |  |  |  |  |  |  |  |  |
| Carew | European | 2003 | 56.6 | 56.0 | 100 | | NS | 124/1449 | 32/333 | 17/1 |  |  |  |  |  | 126/1426 | 30/352 | 1/21 |
| McCarthy | European | 2004 | 48.1 | 43.2 | 70.0 | | 44.0 | 243/312 | 68/82 | 13/4 |  |  |  |  |  |  |  |  |
| Ekstrom | European | 2007 | 52.0 | 53.0 | 83.0 | | 82.0 | 310/323 | 58/62 | 9/2 |  |  |  |  |  |  |  |  |
| Martinelli | European | 2008 | 60.3 | 59.2 | 83.6 | | 66.0 |  |  |  |  |  |  |  |  | 216/116 | 82/57 | 9/9 |
| **subtotal** | **European** |  |  | |  | | | 4357/8184 | 1265/2283 | 98/174 | 61/71 | 242/223 | 293/255 | 10/9 | 5/2 | 691/1836 | 218/548 | 17/42 |
|  |  |  |  |  |  |  |  | **5720/10641** | | | **611/560** | | | | | **926/2426** | | |
| Tamaki | East Asian | 1999 | 59.0 | NS | 76.4 | | NS | 176/245 | 28/38 | 4/2 |  |  |  |  |  |  |  |  |
| Song | East Asian | 2000 | 60.7 | 60.4 | 62.7 | | 61.9 | 140/122 | 18/16 | 0/1 |  |  |  |  |  |  |  |  |
| Cai | East Asian | 2000 | 49.8 | 51.2 | 86.9 | | 82.4 | 125/109 | 12/16 | 0/0 |  |  |  |  |  |  |  |  |
| Tao | East Asian | 2000 | 62.5 | 645.8 | 82.5 | | 67.5 | 109/110 | 11/13 | 0/0 |  |  |  |  |  | 113/116 | 7/6 | 0/1 |
| Shimokata | East Asian | 2002 | 63.4 | 63.6 | 84.0 | | 68.0 | 237/103 | 18/22 | 0/0 | 49/29 | 113/52 | 93/44 | 0/0 |  | 234/106 | 21/19 | 0/0 |
| Kang | East Asian | 2002 | 64.5 | 64.6 | 73.3 | | 51.7 | 55/136 | 4/10 | 1/3 | 10/33 | 40/74 | 9/40 | 0/1 |  |  |  |  |
| Xu | East Asian | 2003 | 61.8 | 62.5 | 77.8 | | 72.9 | 210/178 | 23/30 | 1/2 | 29/34 | 81/96 | 124/80 | 0/0 |  |  |  |  |
| Ogawa | East Asian | 2004 | 43.9 | 43.7 | 100 | | 100 | 117/131 | 10/17 | 0/2 |  |  |  |  |  |  |  |  |
| Zhang | East Asian | 2004 | 73.0 | 68.0 | 81.8 | | 78.4 | 204/101 | 20/14 | 1/1 | 38/26 | 153/57 | 34/32 | 0/1 |  | 210/107 | 13/8 | 2/1 |
| Lu | East Asian | 2005 | >60 | 68.5 | 57.4 | | 59.2 | 94/104 | 13/15 | 1/1 |  |  |  |  |  |  |  |  |
| HuangH ^c^ | East Asian | 2009 | 59.3 | 58.5 | 81.9 | | 59.8 | 368/368 | 40/108 | 12/32 | 72/120 | 212/244 | 132/140 | 4/4 |  | 364/404 | 52/100 | 4/4 |
| HuangH ^d^ | East Asian | 2009 | 57.0 | 54.1 | 79.6 | | 59.8 | 540/536 | 51/57 | 9/11 | 78/83 | 236/276 | 286/245 | 0/0 |  | 440/376 | 152/200 | 8/28 |
| **subtotal** | **East Asian** |  |  | |  | | | 2375/2243 | 248/356 | 29/55 | 276/325 | 835/799 | 678/581 | 4/6 |  | 1361/1109 | 245/333 | 14/34 |
|  |  |  |  |  |  |  |  | **2652/2654** | | | **1793/1711** | | | | | **1620/1476** | | |
| Salazar | Costa Rica | 2006 | 47.0 | 45.0 | 79.0 | | 70.0 | 130/119 | 35/46 | 1/1 | 21/13 | 69/71 | 69/71 | 0/0 | 0/1 |  |  |  |
| Taymaz | Turkey | 2007 | NS | NS | NS | | NS | 82/25 | 32/12 | 4/1 |  |  |  |  |  | 86/29 | 35/9 | 0/0 |
| Pegoraro | Indian | 2005 | M | M | NS | | NS | 100/152 | 79/128 | 16/20 |  |  |  |  |  | 16/28 | 76/119 | 103/153 |
| Sobti | Indian | 2010 | 59.5 | 59.1 | 75.0 | | 66.7 | 110/78 | 102/150 | 88/72 | 16/25 | 121/124 | 160/145 | 3/3 | 0/3 |  |  |  |
| **subtotal** | **Other** |  |  | |  | | | 422/374 | 248/336 | 109/94 | 37/38 | 190/195 | 229/216 | 3/3 | 0/4 | 102/57 | 111/128 | 103/153 |
|  |  |  |  |  |  |  |  | **779/804** | | | **459/456** | | | | | **316/338** | | |
| **total** |  |  |  | |  | | | **9151/14099** | | | **2863/2727** | | | | | **2862/4240** | | |

^a^ 323 in the 5' promoter region, (10-bp insertion in the promoter region (5'FVII)), where allele *A1* corresponds to the absence of the decamer (0) and allele *A2* to its insertion(10).

^b^ NS=not specified, M=matched.

^c^  Study for Chinese Hui population

^d^ Study for Chinese Han population
